# Genome-Wide Identification of the YABBY Gene Family in Maize and Its Expression Analysis Under Low Phosphorus and High Nitrogen Stress

**DOI:** 10.3390/plants14172763

**Published:** 2025-09-04

**Authors:** Feiyan Li, Shuang Li, Litao Yi, Pu Zhao, Chunhong Ma, Xianting Huang, Jiuguang Wang, Chaoxian Liu, Bo Jiao, Xiupeng Mei, Chaofeng Li

**Affiliations:** 1 Maize Research Institute, Southwest University, Chongqing 400715, China; lifeiyan200103@163.com (F.L.); ls18855077417@126.com (S.L.); 13068332389@163.com (L.Y.); 13062355750@163.com (X.H.); wangjiu@swu.edu.cn (J.W.); cauxian@163.com (C.L.); 2 Engineering Research Center of South Upland Agriculture, Ministry of Education, Chongqing 400715, China; 3 Plant Genetic Engineering Center of Heibei Province, Institute of Biotechnology and Food Science, Hebei Academy of Agriculture and Forestry Sciences, Shijiazhuang 050051, China; zhaopu2009@126.com (P.Z.); mch0609@126.com (C.M.); jiaobo1206@163.com (B.J.)

**Keywords:** maize, YABBY, transcription factor, bioinformatics, stress response

## Abstract

YABBY transcription factors (TFs) are key regulators involved in diverse aspects of plant growth, organogenesis, and adaptation to environmental stresses. However, the functional characteristics of YABBY TFs in maize remain largely unexplored. In this study, we systematically identified 12 *YABBY* genes in the maize genome and characterized their gene structures, physicochemical properties, chromosome location, and genomic collinearity. Phylogenetic analysis classified these genes into five subfamilies, with members of each subfamily exhibiting highly conserved exon–intron structures and motif compositions, indicative of potential functional conservation within subfamilies. Cis-regulatory element analysis indicated that *YABBY* genes may be involved in developmental processes, abiotic stress responses, and light-mediated signaling pathways. Moreover, transcriptome sequencing combined with qRT-PCR validation demonstrated that several *YABBY* genes, including *ZmYABBY2*, *ZmYABBY5*, *ZmYABBY8*, and *ZmYABBY9*, are responsive to low-phosphorus and high-nitrogen conditions, implying their potential roles in nutrient stress adaptation. It is worth mentioning that this study redefined the composition of the maize YABBY gene family by excluding a previously annotated member and, for the first time, established a link between YABBY transcription factors and nutrient stress responses. Meanwhile, this is also the first time that protein structure analysis, cis-regulatory element analysis, interspecific collinearity analysis and subcellular localization have been performed on maize ZmYABBY gene family. In summary, our study provides valuable gene resources for maize molecular breeding and offers new insights into the functions of YABBY TFs.

## 1. Introduction

Transcription factors (TFs) are proteins that control chromatin structure and the transcription process by recognizing specific DNA sequences, thereby forming a complex system that guides genome expression [1]. TFs are involved in regulating many processes via the transcriptional regulatory networks and play a vital role in controlling growth and development in organisms [2]. Recently, the Plant Transcription Factor Database v5.0 (PlantTFDB, https://planttfdb.gao-lab.org/, accessed on 21 December 2024) currently contains genomic data for approximately 58 TF families across 165 plant species [3]. Among them, the YABBY gene family is a plant-specific TF family belonging to the zinc finger superfamily. Studies have found that the members of YABBY family play key roles in regulating plant morphogenesis, development processes, phytohormone-mediated signaling pathways, and responses to biotic and abiotic stresses [4].

A defining feature of YABBY family members is the presence of a highly conserved C2C2 zinc finger domain at the N-terminus and a helix–loop–helix (HLH) YABBY domain at the C-terminus. Both domains have been implicated in sequence-specific DNA binding, thereby underpinning the transcriptional regulatory functions of these proteins [5]. Although the YABBY gene family is present in a wide range of plant species, it is relatively small in size, with only a limited number of members identified across species. A total of six members have been identified in *Arabidopsis thaliana* [6], six in pomegranate (*Punica granatum*) [7], eight in common bean (*Phaseolus vulgaris*) [8], and seven in milkvetch (*Astragalus membranaceus*) [9].

YABBY genes have been extensively studied for their roles in plant development and responses to environmental stresses. In *A*. *thaliana*, the family comprises six members: CRC, FIL, YAB2, YAB3, INO, and YAB5 [10]. Among them, CRC and INO are specifically expressed in floral organs [11,12], while FIL, YAB2, and YAB3 are predominantly expressed in the abaxial domains of all leaf-derived organs, including cotyledons, true leaves, and floral organs [10,12]. In tea plant (*Camellia sinensis*) and oil tea (*Camellia oleifera*), CsYABBY7 and CoYABBY3 exhibit flower-specific expression patterns [13]. In grape (*Vitis vinifera*), VvYABBY4 has been implicated in seed development, particularly in endosperm cell growth [14].

YABBY TFs are also involved in phytohormone-mediated signaling and abiotic stress responses. In *Oryza sativa*, OsYABBY4 has been associated with the gibberellin (GA) signaling cascade, modulating downstream gene expression during plant growth and development, whereas OsYABBY1 functions as a negative regulator of GA biosynthesis and metabolism. Ectopic overexpression of OsYABBY1 results in a semi-dwarf phenotype, underscoring its role as a key negative regulator of plant height [15,16].

Maize (*Zea mays* L.), a member of the *Poaceae* family and *Zea* genus, is a major cereal and forage crop with high yield potential and significant economic value. It serves as an essential resource for food, feed, and industrial applications. Although the YABBY transcription factor family in maize has been investigated in previous studies [17,18], the continuous improvement of research methodologies and the progressive updates of genomic databases across plant species call for a re-evaluation of the maize YABBY gene family.

In this study, building upon previous findings, we utilized updated genomic data from publicly available databases and applied comprehensive bioinformatics approaches to identify 12 *YABBY* genes in maize. We conducted a series of analyses, including gene structure characterization, three-dimensional (3D) protein modeling, conserved motif composition, chromosomal localization, phylogenetic analysis, and cis-regulatory element prediction. Furthermore, qRT-PCR validation was performed to correlate the gene expression analysis of *YABBY* under low-phosphorus and high-nitrogen stress with transcriptomic data. Here, the ZmYABBY4 in previous studies [18] has been excluded from the YABBY gene family due to lack of YABBY domain. And the sequences of ZmYABBY1 and ZmYaBBY2 differ by several amino acids with previous version, while the ZmYABBY11 (ZmYABBY12 in previous version) missed 102 amino acids. We firstly performed the protein structure analysis, cis-regulatory element analysis, interspecific collinearity analysis and subcellular localization on maize ZmYABBY gene family and established the link of YABBY nutrient stress adaption. These results provide some useful information for future evolutionary and functional studies of the YABBY gene family in maize.

## 2. Results

### 2.1. Identification and Physicochemical Characterization of Maize YABBY Gene Family Members

Based on amino acid sequences retrieved from the PlantTFDB database (accessed on 21 December 2024), a BLAST search was performed against the maize genome from Phytozome v13 (the Plant Comparative Genomics portal of the Department of Energy’s Joint Genome Institute, United States Department of Energy, Washington, USA, accessed on 24 December 2024) to identify homologous sequences. After removing redundant entries, 12 non-redundant *YABBY* gene candidates were confirmed through conserved domain identification, motif analysis, and phylogenetic relationship evaluation. These genes were designated *ZmYABBY1* to *ZmYABBY12* according to their chromosomal positions.

Physicochemical analysis (Supplementary Table S1) revealed that the predicted YABBY proteins ranged in length from 169 amino acids (aa) (ZmYABBY9) to 320 aa (ZmYABBY7). The molecular weight (MW) ranged from 18.57 kDa (ZmYABBY10) to 33.40 kDa (ZmYABBY3), while the theoretical isoelectric point (pI) varied between 6.22 (ZmYABBY4) and 9.15 (ZmYABBY9).

Subcellular localization predictions using the CELLO v2.5 tool (https://cello.life.nctu.edu.tw/, accessed on 6 January 2025) indicated that all 12 ZmYABBY proteins are predominantly localized in the nucleus, with three members (ZmYABBY2, ZmYABBY5, and ZmYABBY10) potentially dual-localized in both the nucleus and extracellular region.

The instability index of all ZmYABBY proteins exceeded 40, suggesting they are unstable proteins. The aliphatic index ranged from 62.84 to 79.07, indicating moderate thermostability. According to the grand average of hydropathicity (GRAVY), proteins with positive values are considered hydrophobic, those with negative values are hydrophilic, and values between −0.5 and 0.5 represent amphipathic proteins. The GRAVY values of ZmYABBY proteins ranged from −0.54 to −0.18, suggesting that most are hydrophilic proteins, with three members classified as amphipathic proteins (Supplementary Table S1).

Signal peptide prediction using SignalP indicated that none of the ZmYABBY proteins contain signal peptides, suggesting they are not directed to the secretory pathway and are unlikely to be involved in transmembrane transport.

### 2.2. Chromosomal Localization and Protein Structure Analysis of Maize YABBY Family Members

**Figure 1:**
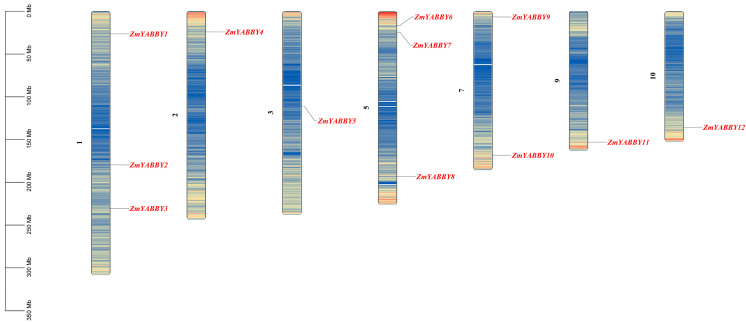
Chromosomal localization of YABBY family members in maize. The lines indicate chromosomal positions harboring *YABBY* genes, and the gene names in red correspond to the respective *YABBY* loci. The number to the left of the chromosome indicates the chromosome number.

Chromosomal mapping and visualization of the 12 maize YABBY gene family members revealed an uneven distribution across chromosomes. Chromosomes 1 and 5 harbored the highest number of *YABBY* genes, with three members each. Chromosome 7 contained two members, while chromosomes 2, 3, 9, and 10 each contained one *YABBY* gene (Figure 1).

Secondary structure prediction of the YABBY proteins revealed that all 12 members contain α-helices, extended β-strands, and random coils, whereas β-turns are entirely absent. Among these structural elements, random coils are predominant, comprising approximately 75.93% of the secondary structure (Supplementary Table S2), indicating that disordered coil regions largely define the secondary structural architecture of maize YABBY proteins.

**Figure 2:**
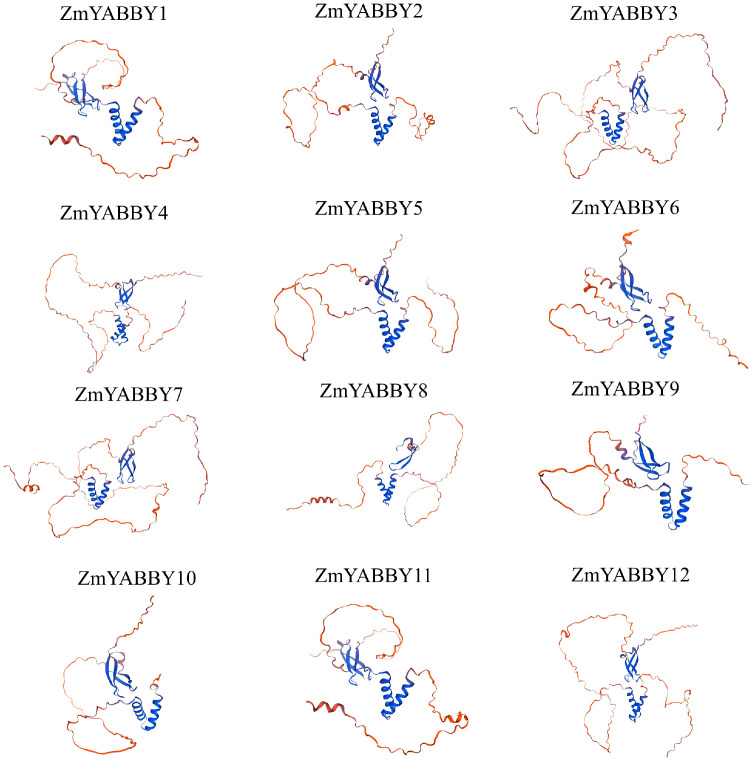
Predicted 3D structures of maize YABBY proteins.

Tertiary structure modeling of the ZmYABBY proteins was conducted using the SWISS-MODEL online tool (https://swissmodel.expasy.org/, accessed on 18 January 2025). The results demonstrated that proteins within the same subfamily exhibit highly similar 3D conformations—for example, ZmYABBY2, ZmYABBY5, ZmYABBY6, and ZmYABBY9 (all in the YAB2 subfamily) share comparable structural features. Likewise, proteins grouped in the same phylogenetic clade—such as ZmYABBY1 and ZmYABBY11, ZmYABBY3 and ZmYABBY7, ZmYABBY4 and ZmYABBY12, and ZmYABBY2 and ZmYABBY5—exhibited nearly identical 3D structures (Figure 2). Apart from ZmYABBY8, which lacked a portion of its domain, the remaining proteins displayed highly conserved spatial configurations of both the HLH YABBY domain and the C2C2-type zinc finger domain. These findings further support the conclusion that the tertiary structures of all maize YABBY proteins are mainly composed of α-helices, extended strands, and random coils, with no presence of β-turns (Figure 2).

### 2.3. Phylogenetic and Multiple Sequence Alignment Analysis of the Maize YABBY Gene Family

**Figure 3:**
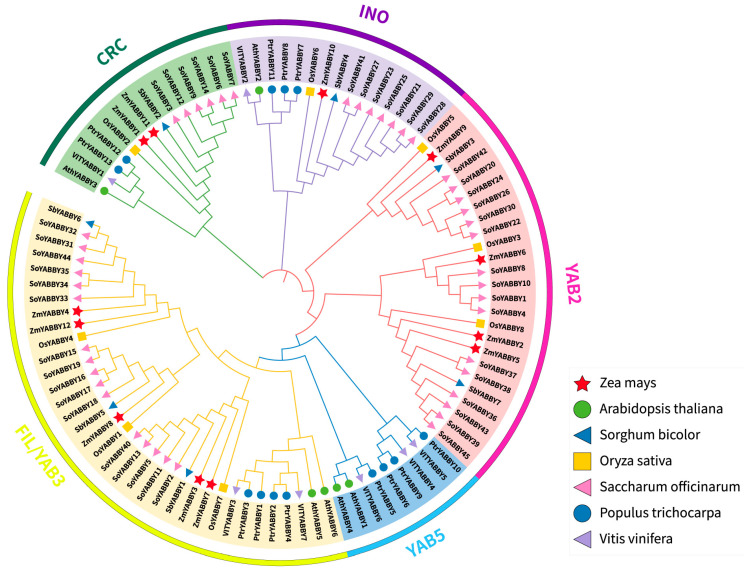
Phylogenetic tree of YABBY proteins from seven species. The outer circle of the phylogenetic tree indicates the subfamily classification of the YABBY genes, the middle circle displays the names of the corresponding YABBY genes, and the inner circle represents the specific clades to which each gene belongs. The shapes on the right denote the species origin of the genes. *Zea mays* (Zm), *Arabidopsis thaliana* (Ath), *Oryza sativa* (Os), *Sorghum bicolor* (Sb), *Saccharum officinarum* (So), *Vitis vinifera* (Vv), and *Populus trichocarpa* (Ptr).

To elucidate the evolutionary relationships among *YABBY* genes in maize, a NJ tree was constructed using MEGA software (Version 11.0.13) with 1000 bootstrap replicates. The analysis included a total of 98 YABBY protein sequences, comprising 6 *A. thaliana*, 8 *O. sativa*, and other representative YABBY family members from diverse plant species (Figure 3).

**Figure 4:**
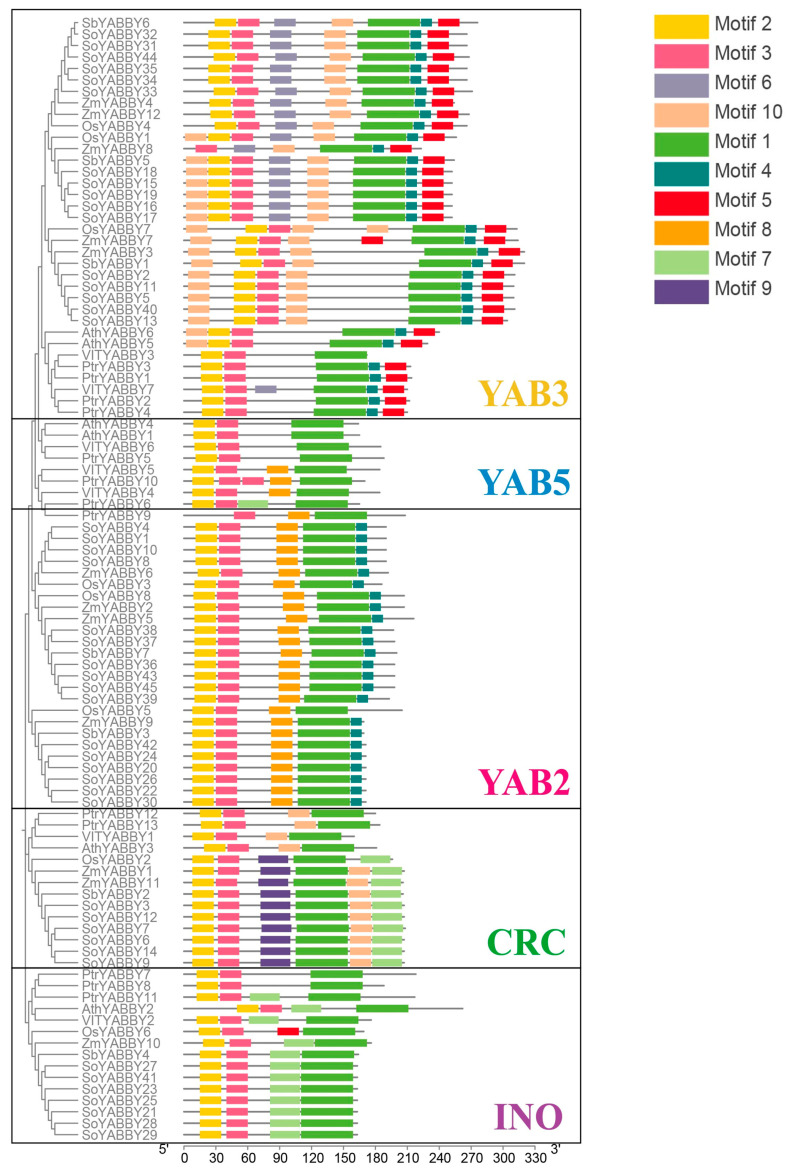
Conserved motif analysis of YABBY family members across seven species. The color-coded module on the right represents the conserved domains present within the YABBY genes.

Based on the phylogenetic relationships of *Arabidopsis* YABBY family members [19] and the conserved motif composition of YABBY proteins across seven species (Figure 4), the 12 maize YABBY genes were classified into five distinct subfamilies: FIL/YAB3, YAB5, YAB2, CRC, and INO.

Among the five subfamilies, FIL/YAB3 and YAB2 represent the largest groups, comprising five (ZmYABBY3, ZmYABBY4, ZmYABBY7, ZmYABBY8, and ZmYABBY12) and four (ZmYABBY2, ZmYABBY5, ZmYABBY6, and ZmYABBY9) members, respectively. The CRC subfamily includes two members (ZmYABBY1 and ZmYABBY11), while the INO subfamily contains a single member (ZmYABBY10). Notably, no maize YABBY gene was assigned to the YAB5 subfamily.

Furthermore, the phylogenetic analysis revealed a close evolutionary relationship between maize and rice YABBY genes, which also share strong homology with those of sugarcane and sorghum—a pattern consistent with their classification as monocotyledonous plants. In contrast, YABBY genes from dicotyledonous species, such as grape (*V. vinifera*), poplar (*P. trichocarpa*), and *Arabidopsis*, clustered more closely with each other, reflecting their evolutionary divergence from monocots.

**Figure 5:**
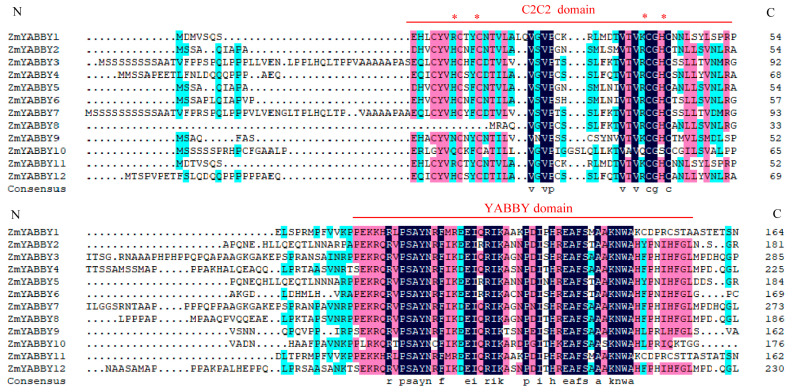
Multiple sequence alignment of maize YABBY proteins. Sequences were aligned with the software of DNAMAN 8. Number on the left side refers to its sequence positions. The C2C2 and YABBY domain repeats are indicated with red lines. The asterisk indicates the location of cysteine in the C2H2domain. Blue (50%), pink shades (75%), and dark purple (100%) are used to show the identification and similarities of the amino acid residues, respectively.

Multiple sequence alignment of the maize YABBY gene family was performed using DNAMAN 8 software with default parameters. The alignment results revealed that most ZmYABBY proteins possess two conserved domains: an N-terminal C2C2 zinc finger domain and a C-terminal YABBY domain (Figure 5). Notably, ZmYABBY8 exhibits a partial truncation in the N-terminal region of the zinc finger domain, whereas the YABBY domain remains completely conserved.

### 2.4. Gene Structure and Conserved Domain Analysis of the Maize YABBY Gene Family

**Figure 6:**
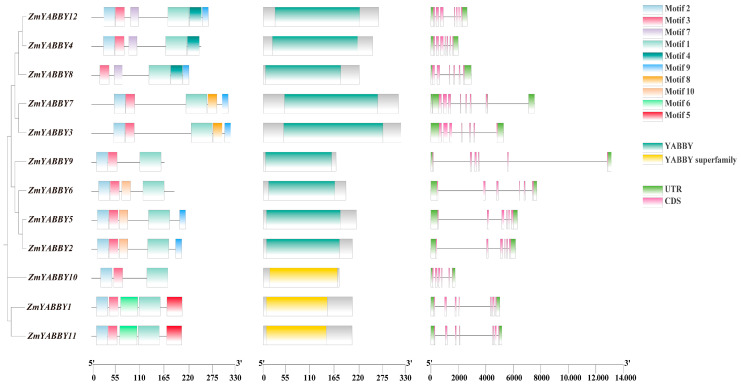
Conserved motifs and gene structure of maize YABBY family members. The left panel depicts the conserved amino acid motifs within the YABBY genes, the middle panel represents the YABBY domains, and the right panel illustrates the gene structures, including coding sequences (CDSs), introns, and untranslated regions (UTRs).

To investigate the gene structure of YABBY family members in maize, ten conserved motifs were predicted using the MEME Suite, and conserved domains were identified through NCBI’s CD-Search tool (https://www.ncbi.nlm.nih.gov/Structure/cdd/wrpsb.cgi, accessed on 11 February 2025). The exon–intron architecture was visualized using TBtools-II (Version 2.311, Figure 6).

From the gene structure analysis, all maize *YABBY* genes contain complete coding sequences (CDSs), with most genes harboring 6 to 7 exons. Notably, *ZmYABBY1* and *ZmYABBY11*, as well as *ZmYABBY2* and *ZmYABBY5*, exhibit highly similar exon–intron arrangements. All members of the YAB2 subfamily share a conserved structure of six exons and five introns, while members of the CRC subfamily possess seven exons and six introns. Most members of the YAB3 subfamily (with the exception of *ZmYABBY8*, which contains seven exons and seven introns) have seven exons and six introns.

All maize YABBY genes encode proteins containing the characteristic YABBY domain. Motif analysis revealed that each ZmYABBY protein contains between three to six conserved motifs, and motifs are highly conserved within the same subfamily. For instance, motif 5 and motif 6 are exclusively present in members of the CRC subfamily, while motif 7 and motif 8 are specific to YAB3 subfamily members. Most ZmYABBY proteins universally possess motif 1, motif 2, and motif 3, in which motif 2 and motif 3 together constitute the N-terminal C2C2-type zinc finger domain, while motif 1 encodes the YABBY domain. Notably, motif 2 is absent in ZmYABBY8, consistent with the multiple sequence alignment results indicating a deletion within its N-terminal zinc finger domain.

### 2.5. cis-Regulatory Element Analysis of Maize YABBY Gene Promoters

**Figure 7:**
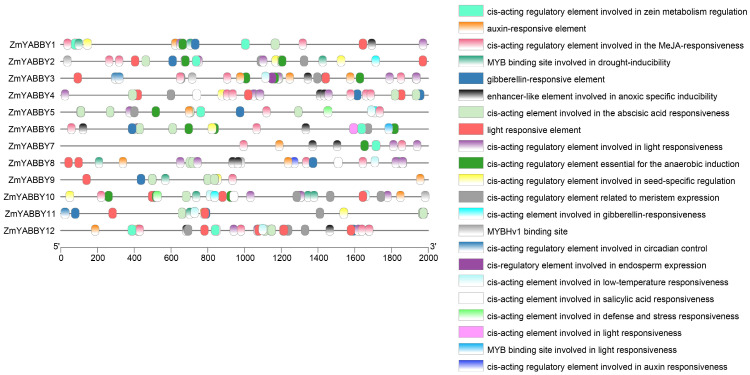
Cis-regulatory elements in the promoter regions of maize YABBY family genes. The right panel shows the cis-regulatory elements present in the promoter regions, while the left panel indicates their positional distribution within the YABBY gene promoters.

To explore the regulatory potential of maize *YABBY* gene promoters, cis-regulatory elements were predicted using online tools and visualized accordingly. The analysis revealed that the promoter regions of maize *YABBY* genes harbor a diverse array of cis-regulatory elements (Figure 7). These predicted elements are primarily associated with phytohormone responsiveness, light responsiveness, drought stress, and low-temperature response.

All maize *YABBY* promoters contain light-responsive cis-regulatory elements and abscisic acid (ABA)-responsive elements, whereas all members except *ZmYABBY11* also possess methyl jasmonate (MeJA)-responsive elements. *ZmYABBY10* exhibits the greatest diversity of cis-elements, with 15 distinct types identified, including elements associated with defense and stress responses, meristem-specific expression, cold stress responsiveness, and gibberellin (GA) and auxin signaling. In contrast, *ZmYABBY7* harbors the fewest cis-elements, with only seven predicted elements (Figure 7). *ZmYABBY4*, *ZmYABBY8*, *ZmYABBY10*, and *ZmYABBY12* carry salicylic acid (SA)-responsive elements. *ZmYABBY3* contains a cis-element associated with endosperm-specific expression, while *ZmYABBY3* and *ZmYABBY11* contain circadian rhythm-related cis-elements. Collectively, these findings suggest that the YABBY gene family may play important roles in maize development, abiotic stress responses, and phytohormonal regulation.

### 2.6. Synteny Analysis of the Maize YABBY Gene Family

**Figure 8:**
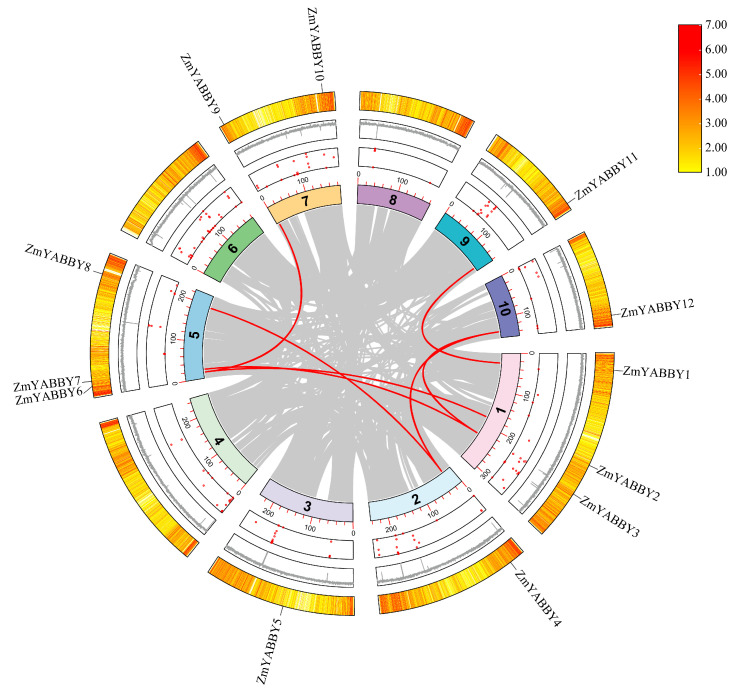
Synteny analysis of YABBY family genes in maize. The gene names in black of outer circle correspond to the respective YABBY loci. Gray lines in the inner circle indicate syntenic gene pairs, while bold red lines represent duplicated gene pairs within the maize YABBY gene family. The number in the inner circle indicates the chromosome number.

To investigate gene duplication events within the ZmYABBY gene family, a synteny analysis was conducted using the MCScanX toolkit. The results identified seven pairs of YABBY genes located within syntenic regions. The outermost ring of the figure represents gene density, which also confirms the chromosomal localization of the ZmYABBY genes, consistent with previous findings. The red dots in the inner circle indicate the number of undetermined bases (N) on each chromosome, corresponding to the fluctuation in the central gray histogram representing genomic complexity (Figure 8).

Notably, most of the syntenic gene pairs occur between members that are closely related on the phylogenetic tree or belong to the same subfamily. For example, within the YAB3 subfamily, *ZmYABBY4* and *ZmYABBY12* exhibit strong synteny. In the YAB2 subfamily, syntenic relationships are observed between *ZmYABBY9* and *ZmYABBY6*, as well as *ZmYABBY2* and *ZmYABBY6*. In the CRC subfamily, a clear syntenic relationship exists between *ZmYABBY1* and *ZmYABBY11* (Figure 8). These gene pairs not only share synteny but also cluster within closely related clades in the phylogenetic tree and exhibit similar conserved motif compositions, suggesting they may have originated from segmental duplications and retained similar functional roles during maize evolution.

**Figure 9:**
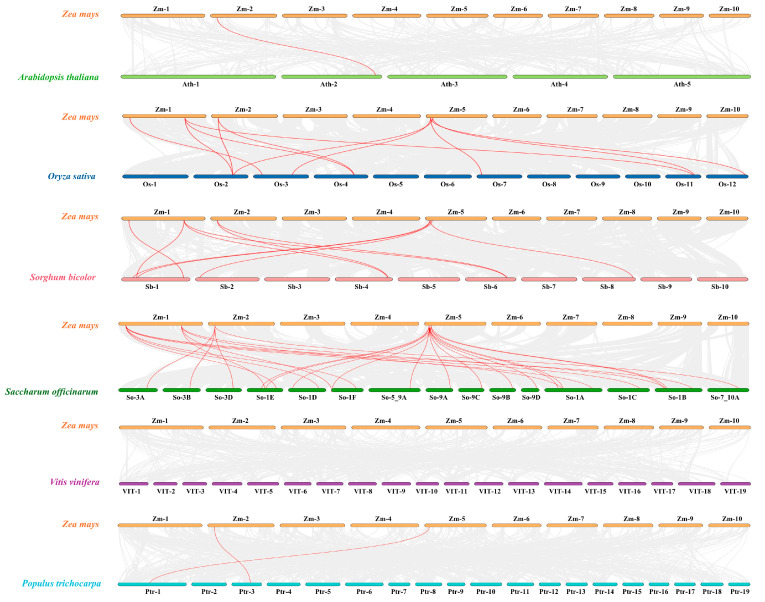
Synteny analysis of *YABBY* family genes between maize and other plant species. Gray lines indicate syntenic gene pairs between maize and *Arabidopsis thaliana* (*Oryza sativa*, *Sorghum bicolor*, *Saccharum officinarum*, *Vitis vinifera*, or *Populus trichocarpa*), while bold red lines represent co-syntenic gene pairs of the *YABBY* gene family between maize and other plant species.

A comparative synteny analysis of YABBY family members was also performed between maize (*Zea mays*) and six other plant species (Figure 9). The results revealed that *ZmYABBY* genes share relatively low synteny with *A. thaliana* (*AthYABBY*) and *P. trichocarpa* (*PrtYABBY*), with only 1–2 syntenic gene pairs detected. Meanwhile, a stronger syntenic relationship was observed between *ZmYABBY* and *OsYABBY* (11 gene pairs) and *SbYABBY* (10 gene pairs). The highest degree of collinearity was found between *ZmYABBY* and *SoYABBY* (sugarcane *YABBY* genes), with 27 syntenic gene pairs identified. Notably, no collinearity was detected between *ZmYABBY* and *VvYABBY* (*V. vinifera YABBY* genes). These results reveal that maize exhibits relatively limited syntenic conservation with woody dicotyledonous species such as grapevine and poplar, while maintaining stronger evolutionary affinities with monocotyledonous species, particularly within the *Poaceae* family. Notably, sugarcane shares the greatest number of syntenic *YABBY* gene pairs with maize, indicating a closer phylogenetic relationship and implying that their *YABBY* gene families may have originated from a common ancestral lineage.

### 2.7. Gene Expression Analysis of YABBY Family Members in Maize

In our previous studies [20,21], maize inbred lines 082 and 107 have been demonstrated to has different phosphorus use efficiency, and inbred line B73 provided the highest quality reference genome. Therefore, to elucidate the expression of maize YABBY genes under nutrition stress, the three maize inbred lines were selected for germination experiments. The seeds were germinated on filter paper moistened with demineralized water. When the seedlings reached the three-leaf stage, uniformly growing plants were selected, roots were gently rinsed, and the seedlings were transferred to Hoagland nutrient solution for low-phosphorus (5 μM KH_2_PO_4_) or high-nitrogen (5 mM KNO3) treatments. After 14 days of treatment, the seedlings were immediately frozen in liquid nitrogen and extracted total RNA for sequencing analysis.

**Figure 10:**
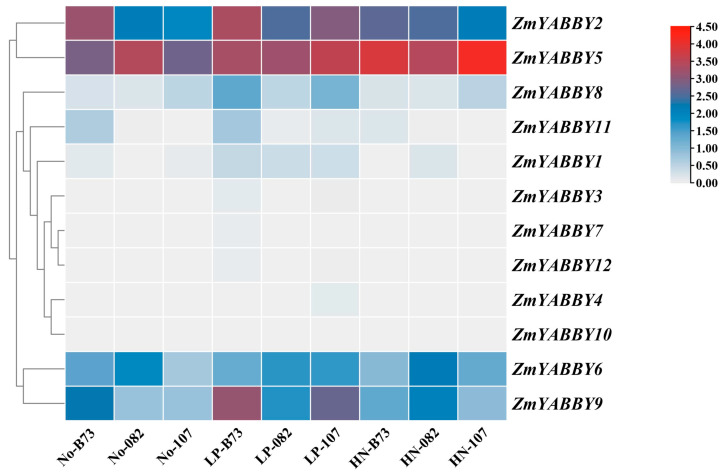
Heatmap of expression levels of maize *YABBY* family members under low phosphorus and high nitrogen treatments. No refers to maize seedling samples grown under normal culture conditions, LP refers to seedlings grown under low-phosphorus conditions, and HN refers to seedlings grown under high-nitrogen conditions. Gray, blue and red boxes indicate low, middle, and high expression levels, respectively.

A heatmap was generated based on normalized transcriptomic data to visualize the expression profiles of maize *YABBY* family members under low-phosphorus (LP) and high-nitrogen (HN) treatments. The results revealed that *ZmYABBY2*, *ZmYABBY5*, *ZmYABBY6*, *ZmYABBY8*, and *ZmYABBY9* had higher expression levels than other genes. *ZmYABBY5* exhibited high expression levels in three inbred lines under any conditions, *ZmYABBY2* was significantly induced by LP and HN treatments in B73 and 082 inbred lines. Meanwhile, the expression of *ZmYABBY6* was not influenced by LP and HN treatments in all three inbred lines. The expression of *ZmYABBY8* and *ZmYABBY9* was induced by LP treatment in B73 and 107 inbred lines. Among the five responsive genes, four (excluding *ZmYABBY5*) showed consistently upregulated expression under both LP and HN treatments compared to the control conditions (Figure 10).

**Figure 11:**
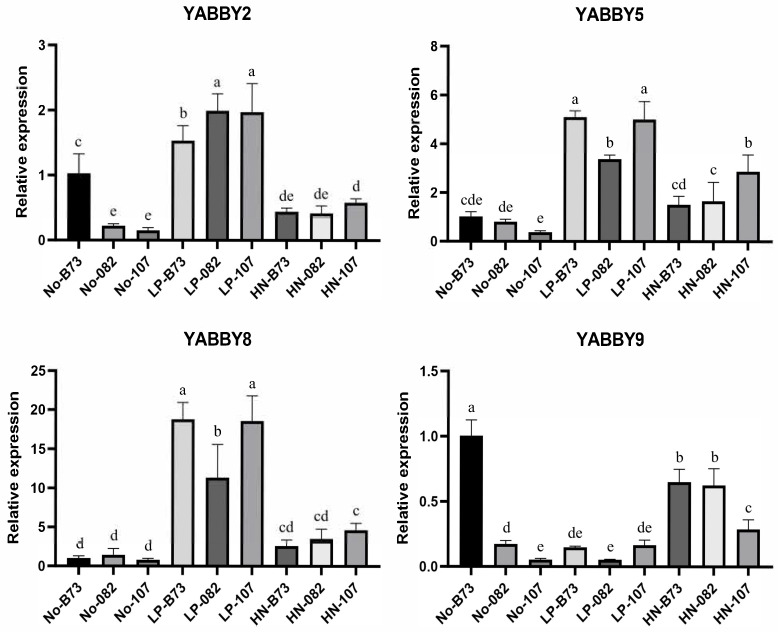
Relative expression levels of four maize *YABBY* genes with qRT-PCR analysis. No refers to maize seedling samples grown under normal culture conditions, LP refers to seedlings grown under low-phosphorus conditions, and HN refers to seedlings grown under high-nitrogen conditions. Different letters above bars represent statistically significant differences between groups (*p* < 0.05) as determined by one-way ANOVA followed by the LSD test and Waller–Duncan’s test.

To further validate the transcriptomic data and elucidate the expression characteristics of *YABBY* genes, qRT-PCR analysis was performed on four differentially expressed *YABBY* genes. In particular, the expression of *ZmYABBY2* was highly induced in three maize inbred lines (B73, 082, and 107) under LP condition, with expression levels notably higher than those under normal nutrient conditions (Figure 11). *ZmYABBY5* and *ZmYABBY8* displayed similar expression patterns and exhibited high expression levels under LP treatment in all three inbred lines. The expression of *ZmYABBY9* was induced by HN treatment in 082 and 107 inbred lines. Generally, although certain differences were observed under specific conditions, the trendy of qPCR results was basically consistent with the RNA-seq data. These findings suggest that specific *ZmYABBY* genes may be involved in the nutrient stress response during maize development, particularly under phosphorus deficiency and nitrogen enrichment.

### 2.8. Subcellular Localization Analysis of ZmYABBY Proteins

The subcellular localization of proteins encoded by ZmYABBY family genes was predicted using CELLO v.2.5, and the results indicated that all ZmYABBY proteins are predominantly localized in the nucleus. To experimentally validate these predictions, the CDSs of two representative genes, ZmYABBY2 and ZmYABBY5, were amplified using high-fidelity DNA polymerase and subsequently cloned into expression vectors carrying green fluorescent protein (GFP) for fusion protein expression. These constructs were transiently expressed in maize protoplasts.

**Figure 12:**
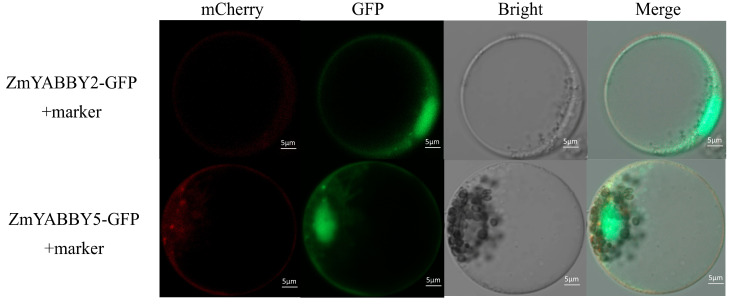
Subcellular localization of proteins encoded by ZmYABBY2 and ZmYABBY5 in maize protoplasts. Bright field and fluorescent micrographs show the localization of ZmYABBY2-GFP and ZmYABBY5-GFP expressed in maize protoplasts of Mo17 inbred lines. mCherry was used as the plasma marker. Scale bars = 5 µm.

As shown in Figure 12, the GFP fluorescence signal from the ZmYABBY-GFP fusion proteins was predominantly localized in the nucleus, consistent with the in silico predictions. Interestingly, ZmYABBY5 also exhibited partial GFP signal at the plasma membrane, where it overlapped with the red fluorescence signal of a membrane marker. This observation suggests that while ZmYABBY proteins primarily function in the nucleus, certain members such as ZmYABBY5 may also localize to the plasma membrane, potentially participating in membrane-associated regulatory functions.

## 3. Discussion

The YABBY gene family plays crucial roles in plant leaf and floral organ development, abiotic stress responses, and lateral organ formation. While *YABBY* genes have been extensively studied in *Arabidopsis* [6], rice [7], switchgrass [22], and tomato [23], relatively few studies have focused on this gene family in maize (*Zea mays*). To provide a systematic understanding of *YABBY* genes in maize, we performed a comprehensive analysis of 12 *ZmYABBY* gene family members based on the B73 reference genome, aiming to gain deeper insights into the structural and functional characteristics of this gene family in maize. Compared with previous studies [17,18], the number of *YABBY* family members decreased by one, and the sequences of several members (*ZmYABBY1*, *ZmYABBY1*, and *ZmYABBY11*) also differ from the previous version. This should be caused by the development of long-read deep sequencing technologies and whole genome assembly of B73 version 5. The results also show the importance of revising the gene family information with the updating databases and genome.

Here, we investigated the basic features of the ZmYABBY gene family. The predicted ZmYABBY proteins range from 169 to 320 amino acids, which is comparable to the YABBY proteins reported in other species such as *Arabidopsis* and Platycodon [24]. Physicochemical property analysis revealed that the average GRAVY values of the ZmYABBY proteins were negative, suggesting they are predominantly hydrophilic—a feature consistent with findings by Huang et al. [25]. The hydrophilic nature of these proteins may contribute to improved abiotic stress tolerance, possibly explaining their roles in environmental stress adaptation. Multiple sequence alignment confirmed that all ZmYABBY proteins contain two conserved domains: the C2C2 zinc finger domain at the N-terminus and the YABBY domain at the C-terminus, indicating high annotation accuracy. These conserved features are consistent with YABBY proteins characterized in other species such as *Arabidopsis* and *Moso bamboo* [26]. Notably, the instability index of these ZmYABBY proteins reveals that ZmYABBY proteins play a different role in response to environment stress, which have been confirmed by Huang et al. [25].

To explore the evolutionary relationships of YABBY proteins in maize, a phylogenetic tree was constructed including YABBY proteins from both monocots (rice, sorghum, sugarcane, and maize) and dicots (*Arabidopsis*, grapevine, and poplar). This study also conducted a gene structure analysis of the maize YABBY family, which had not been performed in previous research [17,18]. Based on phylogenetic topology and conserved motif distributions, all YABBY members were categorized into five subgroups as defined in *Arabidopsis*. Notably, the YAB5 subgroup lacked members from monocots, suggesting that this clade may have been lost in monocots during evolution or has undergone functional divergence. The results reveal that the evolution of YABBY TFs differed between monocots and dicots plants and provide a basis for further study of the evolution of the YABBY family. This observation aligns with YABBY family evolution studies in orchid and wheat [27,28]. Genes within the same subgroup exhibited high conservation in gene structure, motif composition, and predicted tertiary structure. For example, *ZmYABBY1* and *ZmYABBY11* shared nearly identical exon-intron architectures and protein structure, implying they may have originated from a gene duplication event. Meanwhile, synteny analysis among species showed that *ZmYABBY* genes share higher collinearity with monocotyledonous species, whereas they exhibit limited or no collinearity with dicotyledonous species, suggesting that functional divergence occurred between monocots and dicots during evolution—consistent with the aforementioned phylogenetic findings.

Functional divergence of YABBY TFs has also been documented across species. In *Phalaenopsis aphrodite* Rchb. f., PeDL1 regulates ovule and gynostemium development [29]; in cucumber, CsCRCG influences fruit length [30]; in maize, YABBY genes may regulate lateral organ growth rather than cell fate determination [31]; OsYABBY1 in rice is involved in stamen and carpel development and modulates the gibberellin (GA) signaling pathway [32,33]; in *Camellia sinensis*, CsFILa and CsFILb modulate leaf morphogenesis [34]; and in pineapple, AcYABBY4 suppresses root growth under salt stress [35].

The cis-regulatory elements, such as enhancers and promoters, are essential to development, and their divergence is a common cause of evolutionary change [36]. However, the ZmYABBY family has not finished the cis-regulatory element analysis in previous studies [17,18]. In the present study, cis-regulatory element analysis of the *ZmYABBY* promoter regions revealed the presence of multiple elements associated with light responsiveness, plant growth and development, hormone signaling, and stress responses. Notably, the promoters were enriched in auxin-responsive elements, MeJA-responsive elements, ABA-responsive elements, and light-responsive elements, suggesting that *ZmYABBY* genes are likely involved in phytohormone-mediated signaling pathways and stress adaptation. The presence of hormone-responsive cis-elements, such as those for ABA, MeJA, auxin, and GA, further supports the potential role of *ZmYABBYs* in maize growth and developmental regulation.

Nitrogen and phosphorus nutrition are crucial for plant growth. A deficiency of available phosphorus has become one of the major factors limiting maize growth and yield formation, whereas the application of high concentrations of nitrogen fertilizer can significantly promote maize growth. Using RNA-seq and qRT-PCR, we further analyzed the expression profiles of *ZmYABBY* genes under HN and LP conditions. The results demonstrated substantial changes in gene expression under these nutrient treatments compared to control conditions. Among them, *ZmYABBY5* showed a strong and consistent response to both high nitrogen and low phosphorus, indicating a potential role in nutrient stress signaling. Unfortunately, we could not design qRT-PCR primers to amplify its specific fragment (100–200 bp) to distinguish ZmYABBY5 with ZmYABBY2 and other ZmYABBY proteins due to their sequence similarities. Overall, these findings imply that *ZmYABBY* genes may participate in the nutritional response pathways in maize.

Finally, subcellular localization assays indicated that in addition to the nuclear localization predicted for ZmYABBY proteins. This is also the first time to analyze the expression of cellular localization of maize YABBY genes. ZmYABBY2 and ZmYABBY5 showed dual localization at the nucleus and plasma membrane, which are consistent with bioinformatics prediction (Supplementary Table S1). The results indicate that ZmYABBY2 and ZmYABBY5 may possess dual localization and membrane-associated regulatory functions associated with the abaxial cell fate [10]. The finding is consistent with observations reported by Zhao et al. [37], adding new insight into the possible functional diversification of YABBY transcription factors in maize.

## 4. Materials and Methods

### 4.1. Source of Genomic Data

The genomic sequence and gene annotation file (GFF3 format) for maize (*Zea mays*) were downloaded from the Phytozome 13 database (https://phytozome-next.jgi.doe.gov/, accessed on 24 December 2024), specifically from the *Zea mays* Zm-B73-REFERENCE-NAM-5.0.55 genome assembly.

Genomic data, gene annotation files, and corresponding amino acid sequences for *A. thaliana* (Araport11), *O. sativa* (v7.0), *Sorghum bicolor* (v5.1), *Saccharum officinarum × spontaneum* (R570 v2.1), *V. vinifera* (v2.1), and *Populus trichocarpa* (v4.1) were also obtained from the Phytozome database. These datasets were used for comparative genomic and phylogenetic analyses.

### 4.2. Identification of YABBY Gene Family Members in Maize

YABBY family members were first queried from the Plant Transcription Factor Database v5.0 (PlantTFDB, https://planttfdb.gao-lab.org/, accessed on 21 December 2024) [38], and their amino acid sequences were downloaded. These sequences were then used to search for homologous genes in the maize genome using the BLAST tool provided by the Phytozome database. Hits with sequence identity >80%, corresponding to the top-ranked gene for each query, were retained. Redundant sequences were removed, and the remaining protein sequences were downloaded for further analysis.

The obtained amino acid sequences were subjected to conserved domain identification using Batch CD-Search on the NCBI platform (https://www.ncbi.nlm.nih.gov/Structure/bwrpsb/bwrpsb.cgi, accessed on 21 December 2024), with an E-value threshold of 0.01. Conserved motif analysis was performed using the MEME Suite (https://meme-suite.org/meme/tools/meme, accessed on 23 December 2024) [39], with the maximum number of motifs set to 10 and all other parameters left at default values. Sequences lacking the YABBY domain were removed from subsequent analysis.

The remaining candidate sequences were further validated by querying the InterPro database (https://www.ebi.ac.uk/interpro/ accessed on 25 December 2024) to obtain their domain accession numbers. The Hidden Markov Model (HMM) profile file of the YABBY domain (PF04690) was downloaded, and an HMM-based search was conducted using the Simple HMM Search tool in TBtools [40], against the maize B73 inbred line protein dataset. Candidate proteins with E-values less than 1 × 10^−10^ were selected. Redundant and non-conforming sequences were removed.

Finally, domain validation was performed using the SMART database (http://smart.embl-heidelberg.de/, accessed on 27 December 2024), and only those sequences that contained a complete YABBY domain were confirmed as bona fide members of the maize YABBY gene family.

### 4.3. Physicochemical Properties and Protein Structure Prediction of Maize YABBY Family Members

The molecular weight (MW) and theoretical isoelectric point (pI) of maize YABBY proteins were predicted using the Compute pI/Mw tool available on the ExPASy server (https://web.expasy.org/compute_pi/, accessed on 3 January 2025). The gene accession numbers, chromosomal locations, start positions, coding sequence (CDS) lengths, protein sequence lengths, and strand orientation of each YABBY gene were retrieved from the Phytozome database based on their annotated sequences.

Subcellular localization was predicted using CELLO v2.5 (http://cello.life.nctu.edu.tw/, accessed on 6 January 2025) [41]. The instability index, aliphatic index, and grand average of hydropathicity (GRAVY) were calculated using TBtools. Signal peptide prediction was performed using the SignalP 5.0 online server (https://services.healthtech.dtu.dk/services/SignalP-5.0/, accessed on 16 January 2025).

The secondary structure of YABBY proteins was predicted using SOPMA (https://npsa.lyon.inserm.fr/cgi-bin/npsa_automat.pl?page=/NPSA/npsa_sopma.html, accessed on 14 March 2025), which provides estimates of α-helices, β-sheets, turns, and random coils. The three-dimensional (3D) structure of each YABBY protein was modeled using the SWISS-MODEL homology modeling platform (https://swissmodel.expasy.org/, accessed on 18 January 2025), providing insights into their potential structural conformations.

### 4.4. Conserved Domain and Gene Structure Analysis of Maize YABBY Family Members

The maize genome sequence and gene annotation file were downloaded from the Phytozome database, along with the amino acid sequences of the YABBY family members. Gene structure visualization, including exon–intron organization, was performed using TBtools.

Based on the above generated phylogenetic tree, conserved motif analysis results, and conserved domain annotations obtained from NCBI, an integrated schematic diagram was constructed using TBtools. This comprehensive visualization facilitates the comparative analysis of gene structure, motif distribution, and domain conservation among maize YABBY genes.

### 4.5. Chromosomal Mapping and Synteny Analysis of Maize YABBY Gene Family Members

Based on the maize gene annotation file, the chromosomal localization of YABBY gene family members was visualized using TBtools. To assess intraspecific synteny, the MCScanX module integrated in TBtools was employed to identify and visualize segmental and tandem duplication events among maize *YABBY* genes.

Additionally, interspecific synteny analysis was conducted between maize and each of the six aforementioned species (*A. thaliana*, *O. sativa*, *S. bicolor*, *S. officinarum × spontaneum*, *V. vinifera*, and *P. trichocarpa*), to identify conserved collinear blocks and explore the evolutionary relationships of YABBY genes across different plant lineages.

### 4.6. Plant Materials and Treatments

Seeds of three maize (*Zea mays* L.) inbred lines—B73, 082, and 107—were used for germination experiments under controlled conditions (growth chamber at 25 °C). The seeds were obtained from the Maize Research Institute of Southwest University (China). We germinated the seeds on filter paper moistened with demineralized water. When the seedlings reached the three-leaf stage, uniformly growing plants were selected, roots were gently rinsed, and the seedlings were transferred to Hoagland nutrient solution (Coolaber Technology Co., Ltd., Beijing, China). The complete Hoagland medium contains 250 μΜ KH_2_PO_4_, 2 mM Ca(NO3)_2_·4H_2_O, 1.25 mM NH_4_NO_3_, 0.1 mM KCl, 0.65 mM K_2_SO_4_, 0.65 mM MgSO_4_, 10.0 mM H_3_BO_3_, 0.5 mM (NH4)_6_MO_7_O_24_, 1.0 mM MnSO_4_, 0.1 mM CuSO_4_·5H_2_O, 1.0 mM ZnSO_4_·7H_2_O, and 0.1 mM Fe-EDTA. According to previous studies, 5 μM KH_2_PO_4_ [21] was used for low-phosphorus treatment, and 5 mM KNO_3_ was used for high-nitrogen treatment [42].

The plants were grown in a growth chamber with a photoperiod of 14 h light/10 h dark, day/night temperatures of 28 °C/24 °C, and 65% relative humidity. After 14 days of treatment, seedlings with uniform growth and similar phenotypic performance were selected. For each treatment, four healthy plants were pooled as a biological replicate, and three biological replicates were collected per condition. The samples were immediately frozen in liquid nitrogen and sent to Biomarker Technologies Co., Ltd. (Beijing, China) for RNA sequencing (RNA-seq) analysis.

### 4.7. Gene Expression Analysis of Maize YABBY Family Members

The expression profiles of YABBY family genes were analyzed based on the FPKM (Fragments Per Kilobase of transcript per Million mapped reads) values provided by the sequencing company. The data were log2-transformed [log2(FPKM + 1)] for normalization and subsequently visualized as a heatmap using the HeatMap function in TBtools.

To validate the transcriptome (RNA-Seq) results, we selected YABBY genes that exhibited relatively high expression levels under both high-nitrogen and low-phosphorus conditions for quantitative real-time PCR (qRT-PCR) analysis.

Total RNA was reverse-transcribed into cDNA using the NovoScript^®^ Plus All-in-one 1st Strand cDNA Synthesis SuperMix (gDNA Purge) kit (E047-01B; Novoprotein, Shanghai, China). Gene-specific primers for qRT-PCR were designed using the Primer-BLAST tool on the NCBI website (https://www.ncbi.nlm.nih.gov/tools/primer-blast/, accessed on 24 December 2024). The *Actin* gene of maize was used as the internal reference.

Each 20 μL qRT-PCR reaction contained 0.5 μL of each forward and reverse primer, 1 μL of cDNA template, 8 μL of nuclease-free water, and 10 μL of 2× NovoStart^®^ SYBR qPCR SuperMix Plus (Cat.No.E096; Novoprotein, Shanghai, China). Each reaction was performed in technical triplicates under the following cycling conditions: initial denaturation at 95 °C for 5 min, followed by 40 cycles of 95 °C for 30 s (denaturation), 60 °C for 20 s (annealing), and 72 °C for 30 s (extension). Reactions were conducted on the Bio-Rad CFX96 Real-Time PCR Detection System (CFX96 Touch, Bio-Rad, Hercules, CA, USA).

Relative expression levels of selected genes were calculated using the 2^−ΔΔCT^ method [43], and results were visualized using GraphPad Prism 5.0. The sequences of primers used in the qRT-PCR assays are listed in Supplementary Table S3.

### 4.8. Subcellular Localization of ZmYABB2/5 Proteins

The subcellular localization of ZmYABBY2/5 proteins was predicted using CELLO v.2.5 (http://cello.life.nctu.edu.tw/, accessed on 6 January 2025). The coding sequences (CDS) of each ZmYABBY gene (excluding the stop codon) were amplified via high-fidelity PCR using 2× Phanta Flash Master Mix (Vazyme, Nanjing, China). The PCR products were cloned into the pAN580 vector, which carries a GFP (green fluorescent protein) tag, using LightNing^®^ DNA Assembly Mix Plus (Yugong Biotech, Lianyungang, China). XbaI and BamHI restriction sites were used to linearize the pAN580-GFP vector for cloning.

The GFP fusion proteins were transiently expressed in maize protoplasts, which were isolated from etiolated seedlings of the Mo17 inbred line, using a PEG-mediated transformation method [44]. Transformed protoplasts were incubated at 23 °C for 14 h in a growth chamber. GFP fluorescence signals were then observed using a laser scanning confocal microscope (LSM900, ZEISS, Baden-Württemberg, Germany). Primer sequences used for construction of the GFP fusion vectors are listed in Supplementary Table S3.

### 4.9. Statistical Analysis

QRT-PCR data were analyzed by one-way ANOVA. When significant differences were detected (*p* < 0.05), pairwise comparisons were performed using the least significant difference (LSD) test, and the Waller–Duncan’s test was additionally applied to confirm the robustness of multiple comparisons. All analyses were conducted in SPSS 27.0 with a significance level of *p* < 0.05. In bar charts, different lowercase letters (e.g., a, b, c) indicate significant differences among groups (*p* < 0.05), whereas identical letters represent non-significant differences (*p* > 0.05).

## 5. Conclusions

In this study, we identified a total of 12 YABBY family members in maize and conducted a comprehensive genome-wide analysis, including phylogenetic relationships, functional classification, gene structure, conserved motif composition, and chromosomal localization. Cis-regulatory element analysis revealed the presence of light-responsive elements and ABA-responsive elements in all ZmYABBY genes, indicating their potential roles in regulating plant growth and development. Furthermore, RNA-seq combined with qRT-PCR analysis revealed significant expression changes in several ZmYABBY genes under high-nitrogen and low-phosphorus treatments, implying that the YABBY gene family in maize may be involved in nutrient-responsive pathways, particularly in nitrogen and phosphorus uptake and signaling. Overall, our findings provide a solid foundation for future functional characterization of ZmYABBY genes and offer valuable insights into their potential roles in maize development and environmental responses.

## Data Availability

All additional datasets supporting the findings of this study are included within the article and Supplementary Materials. The transcriptomes data of low-phosphorus or high-nitrogen treatments have been deposited in the Genome Sequence Archive of the BIG Submission Portal, National Genomics Data Center (NGDC, https://ngdc.cncb.ac.cn/) under the Bioproject (PRJCA044786).

## Supplementary Materials

The following supporting information can be downloaded at https://www.mdpi.com/article/10.3390/plants14172763/s1: Table S1: The detailed information of YABBY members; Table S2: Prediction of Secondary Structure of YABBY Protein; and Table S3: The Primers used in this study.
